# Facile synthesis of two-dimensional Ruddlesden–Popper perovskite quantum dots with fine-tunable optical properties

**DOI:** 10.1186/s11671-018-2664-5

**Published:** 2018-08-22

**Authors:** Yi-Hsuan Chang, Jou-Chun Lin, Yi-Chia Chen, Tsung-Rong Kuo, Di-Yan Wang

**Affiliations:** 1 Department of Chemistry, Tunghai University, Taichung, 40704 Taiwan; 2 Graduate Institute of Nanomedicine and Medical Engineering, College of Biomedical Engineering, Taipei Medical University, Taipei, 11031 Taiwan

**Keywords:** Two-dimensional Ruddlesden–Popper perovskite, Nanocrystal, One-pot synthesis, Quantum confinement effect

## Abstract

In hybrid organic–inorganic and all-inorganic metal halide perovskite nanomaterials, two-dimensional (2D) Ruddlesden–Popper (RP) perovskites have become one of the most interesting materials because of tunable bandgaps varied with the layer thickness, effective modulation of the electron-hole confinement, and high stability. Here, we report a one-pot synthesis of 2D RP perovskite (BA)_2_(MA)_*n −* 1_Pb_*n*_X_3*n* + 1_ (BA = 1-butylammonium, MA = methylammonium, X = Br or I) quantum dots (QDs) with an average size of 10 nm at room temperature. The (BA)_2_(MA)_*n −* 1_Pb_*n*_Br_3*n* + 1_ (Br series) QDs and (BA)_2_(MA)_*n* − 1_Pb_*n*_I_3*n* + 1_ (I series) QDs exhibited tunable emitting spectrum in the range of 410–523 nm and 527–761 nm, respectively, with full width at half maximum (FWHM) of 12–75 nm. The emission color was tuned by the ratio of MA and halide. The photoluminescence quantum yield of 2D perovskite QDs reached 48.6% with more thermodynamic stability in comparison with 3D MAPbX_3_ QDs. Overall results indicated that developing a solution synthesis for 2D RP perovskite QDs with great optical properties paves the way toward future optoelectronic devices and perovskite quantum dot photovoltaics.

**Electronic supplementary material:**

The online version of this article (10.1186/s11671-018-2664-5) contains supplementary material, which is available to authorized users.

## Background

Development of new fluorescent materials with the narrow emissive band and color adjustment is a key technology for the lighting and display technologies with high color performance [1–5]. Colloidal quantum dots (QDs) have been considered as promising candidates because of their unique intrinsic properties, such as tunable color light and higher photoluminescence quantum yields (PLQYs) [2, 4]. Instead of traditional II–VI or III–V semiconductors, new 3D organic–inorganic perovskite [6–10] or inorganic halide perovskite QDs [11–17] with unit cell formula AMX_3_ (A is small organic or inorganic cation ((such as CH_3_NH_3_^+^ or Cs^+^), X is a halogen (Cl^−^, Br^−^ or I^−^), M is a metal (Pb or Sn) that can coordinate to six halides) have been developed. These 3D perovskites exhibited excellent performance in the light-emitting diode and solar energy conversion because of the tunability of wavelength (from 400 nm to 800 nm) and sharp emission (full width at half maximum, FWHM ∼ 20 nm) [14, 18–20]. However, one obstacle to 3D perovskite as active materials in photoelectronic applications is the nonradiative pathways through sub-band defect states, resulting in lower PLQYs and less EL emission [21, 22]. Recently, Ruddlesden–Popper perovskites materials with reduced dimensionality have been discovered to be two-dimensional (2D) perovskite structure which was formed by inserting different large organic cations (R) in the A-site of AMX_3_ slices along the crystallographic planes. These 2D layer perovskite materials have a general chemical formula of (RNH_3_)_2_(CH_3_NH_3_)_*n* − 1_A_*n*_X_3*n* + 1_ and exhibit beneficial layer edge states with no typical trap states, resulting in long PL lifetime, relevant photo-stability, and chemical stability for better optoelectronic devices performance [23–26].

Recently, the 2D and 3D perovskite materials are found to be thick and grain size controllable for a higher exciton binding energy with higher electron-hole capture rate for radiative recombination [26, 27]. Besides, 2D-layered perovskites also possess an optical property of the quantum confinement effect wherein the band gap of the perovskites can be adjusted by the different thickness of the perovskite layer [25]. Several reports indicated that 2D-layered perovskite thin films demonstrated good performance in photovoltaic or light-emitting diode because of long-lived free carriers provided by lower energy states at the edges of the layered perovskites and tunable emission wavelength controlled by the thickness of perovskite [23, 25, 28–30]. Because of the unique properties of the 2D perovskite materials, the corresponding colloidal nanocrystal is attractive to be developed and investigated to understand their optical properties for future highly luminescent and stable colloidal perovskite nanocrystals. For example, a series of quasi-2D lead(II) bromide perovskites with submicron size would exhibit different quantum size confinement effects by using different length organic cation which can tune emission from bright green to blue [31, 32]. Up to date, the study of the optical property of 2D perovskite QDs with the size smaller than 10 nm is few. Therefore, size control of 2D perovskite QDs remains an important issue for further photophysical and optoelectronic properties investigation.

In this report, monodisperse 2D Ruddlesden–Popper perovskite (BA)_2_(MA)_*n* − 1_Pb_*n*_X_3*n* + 1_ (BA = 1-butylammonium, MA = methylammonium, X = Br or I) QDs with an average size of 10 nm have been successfully prepared via a facile method. (BA)_2_(MA)_*n* − 1_Pb_*n*_Br_3*n* + 1_ (Br series) and (BA)_2_(MA)_*n* − 1_Pb_*n*_I_3*n* + 1_ (I series) QDs exhibited tunable emitting spectrum in the range of 410–523 nm and 527–761 nm, respectively. The layered structure of 2D perovskite QDs was confirmed by X-ray diffraction (XRD). Photoluminescence (PL) of 2D perovskite QDs was characterized with a sharp emission (FWHM) of 12–42 nm, high quantum yields of 6.8–48.6%, and short radiative lifetimes of 1.6–75.9 ns.

## Results and discussion

**Fig. 1:**
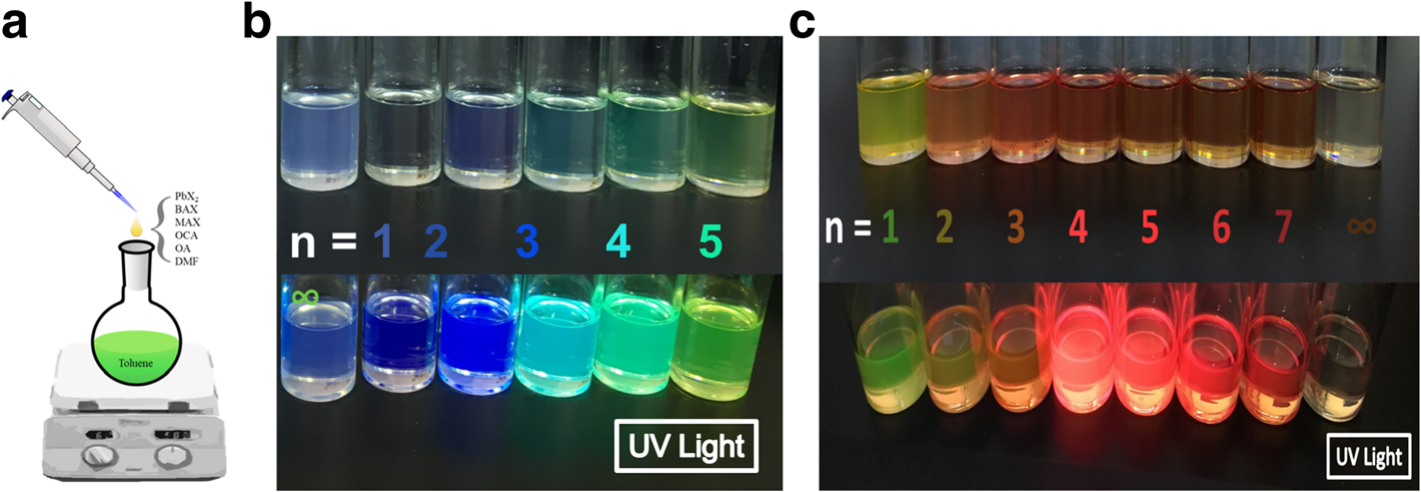
**a** Schematic illustrating the one-pot synthesis process to prepare the 2D RP perovskite QDs at room temperature. The photographs of 2D RP perovskite QDs with **b** Br series and **c** I series dissolved in toluene taken under ambient condition (upper part) and UV light (lower part) (λ = 365 nm)

2D (BA)_2_(MA)_*n* − 1_Pb_*n*_X_3*n* + 1_ perovskite QDs were fabricated by a facile one-pot synthesis method, as shown in Fig. 1a. First, a precursor solution was prepared by dissolving PbX_2_ (X = Br or I), methylammonium halogen (MAX), butylammonium halogen (BAX), octylamine (OLA), and oleic acid (OA) with proper ratios in the dimethylformamide (DMF) solution. The resulting solution was added drop by drop into a quenching solvent chlorobenzene to form 2D perovskite QDs at ambient conditions. By adjusting the ratio of MAX and PbX_2_ in a precursor solution (in Table 1), 2D perovskite QDs with different *n* values will be performed. The OA and OLA played a role in the co-surfactants to stabilize the growth of QDs. The as-prepared 2D Br series and I series perovskite QDs were found to be dispersed well and the corresponding photo-images (Fig. 1b, c) of the QDs with increases of “*n*” value showed the changes of emission color from blue to greenish and greenish to bright red under ultraviolet light irradiation, respectively. Especially, 3D lead (II) iodide perovskite QDs (*n* = ∞) exhibited the weakest emission 2D I series perovskite QDs with other *n* values. The result also showed that the 2D perovskite QDs exhibited more structural and optical stability than 3D perovskite QDs after formation of QDs.

**Table 1:** The lists of synthesis required compositions for 2D RP perovskite QDs

A’_2_A_*n* − 1_B_*n*_X_3*n* + 1_ (X = Br, I)	(BA)X (mmol)	(MA)X (mmol)	PbX_2_ (mmol)	*n*
(BA)_2_PbX_4_	2	0	1	1
(BA)_2_(MA)Pb_2_X_7_	2	1	2	2
(BA)_2_(MA)_2_Pb_3_X_10_	2	2	3	3
(BA)_2_(MA)_3_Pb_4_X_13_	2	3	4	4
(BA)_2_(MA)_4_Pb_5_X_16_	2	4	5	5
(BA)_2_(MA)_*n*-1_Pb_*n*_X_3*n*+1_	2	*n* − 1	*n*	*n*
(MA)PbX_3_	0	5	5	∞

**Fig. 2:**
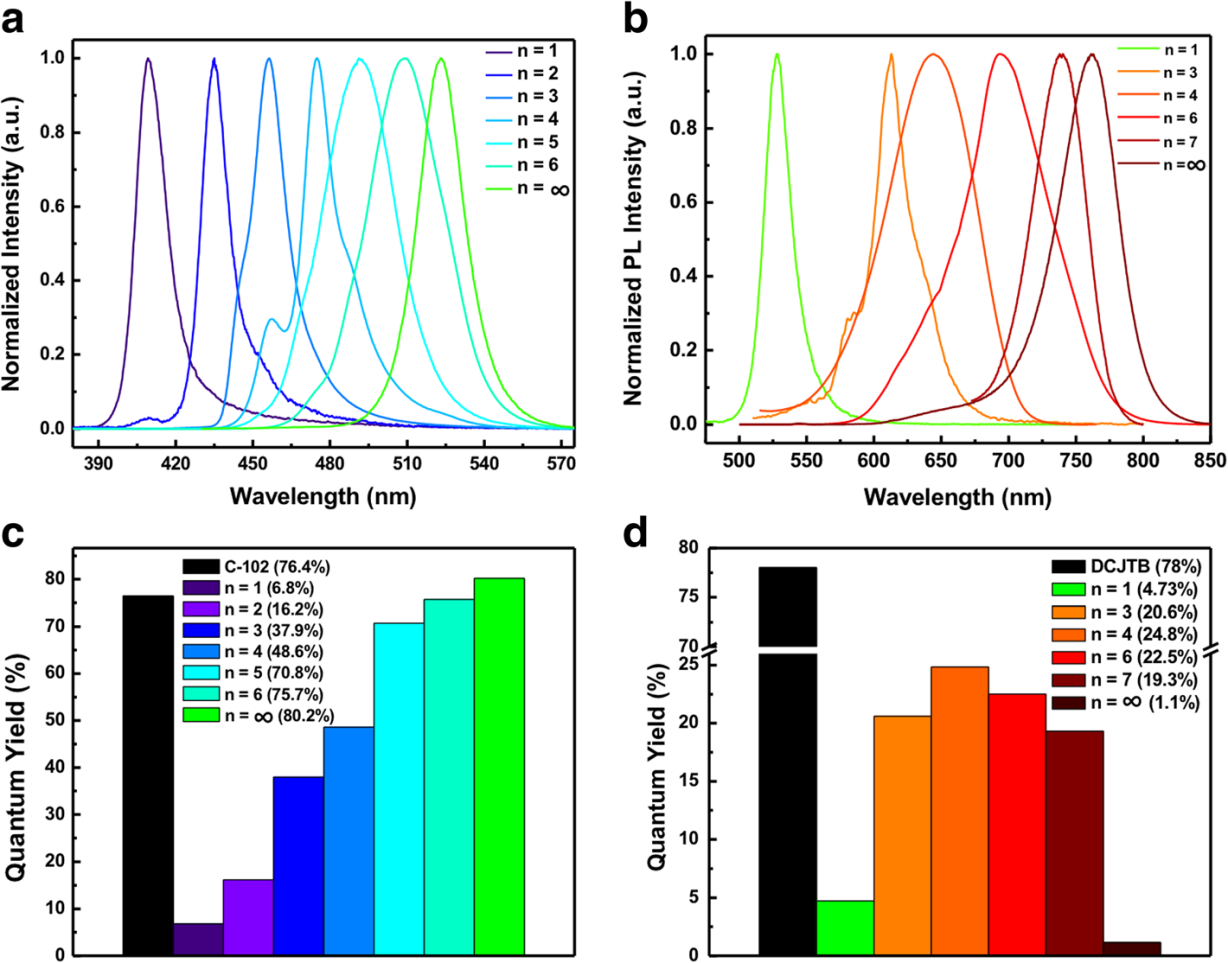
PL emission spectra of 2D RP perovskite QDs with **a** Br series and **b** I series with varied *n* values. The corresponding quantum yield of 2D RP perovskite QDs with **c** Br series and **d** I series

To investigate the optical properties of 2D Br series and I series perovskite QDs with different emission color, the PL spectra of these 2D perovskite QDs in chlorobenzene (CB) solvent were measured as shown in Fig. 2. The PL spectra of 2D perovskite QDs for Br series and I series exhibit emission wavelength across the visible region from 410 to 523 nm and 527–761 nm, respectively. Both PL spectra for two series QDs show a red shift upon increase of *n* value and low FWHM values of each emission around ~ 11–21 nm, suggesting the formation of highly pure 2D perovskite QDs. Br series with *n* = 4 and 5 and I series with *n* = 3 and 4 exhibited a main peak along with a small shoulder attributed to a mixture of 2D perovskite QDs with different *n* values in the same solution. Especially, the emission peak of (BA)_2_(MA)_*n −* 1_Pb_*n*_I_3*n* + 1_ with *n* = 1 at 527 nm is observed, indicating a larger band gap in comparison with previous reports. High PLQYs of 2D Br series and I series perovskite QDs (Fig. 2c, d) were obtained from 6.8 to 48.6% and 1.1 to 24.8%, respectively. Overall results indicated that the 2D perovskite QDs exhibited an obviously quantum confinement effect due to the formation of quantum well by separating different thicknesses of the inorganic layers from the BA molecules as spacers.

**Fig. 3:**
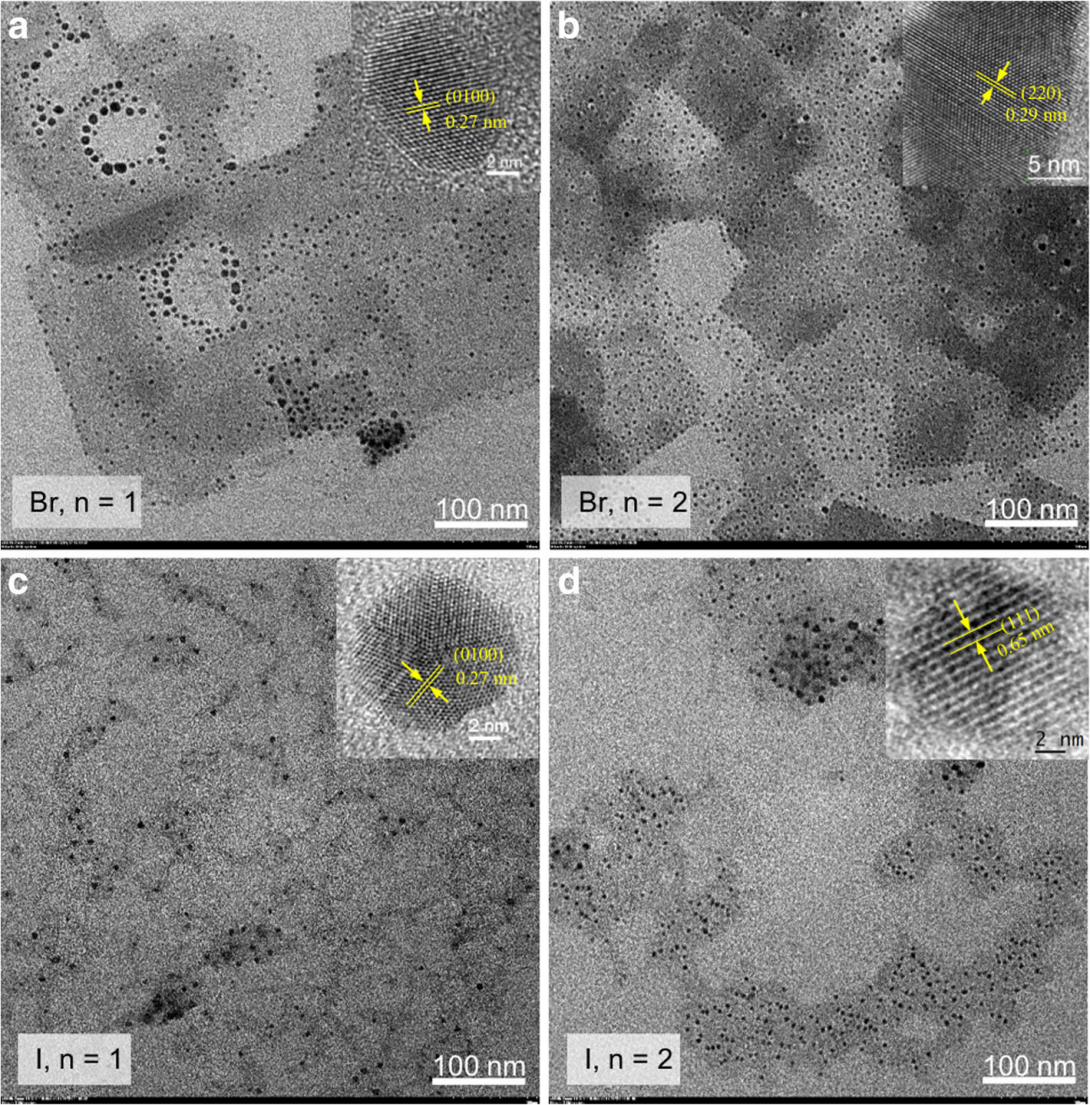
**a**–**b** TEM images of 2D RP perovskite QDs with Br series (*n* = 1 and 2), respectively. **c**–**d** TEM images of 2D RP perovskite QDs with I series (*n* = 1 and 2), respectively. The insets are the HRTEM image of representative 2D RP perovskite QDs

Figure 3 shows that the representative transmission electron microscopy (TEM) images of well-dispersed 2D Br series and I series perovskite QDs with *n* = 1 and 2 exhibited spherical shape with a small size distribution. The average size of these QDs is around 10 nm. The results indicate that the crystal growth of the QDs is controlled by co-surfactants (OA and OLA). Besides, other 2D perovskite QDs with different *n* values are shown in (Additional file 1: Figure S1). The representative high-resolution TEM (HRTEM) images (the inset of Fig. 3a–d) show clear lattice structures of the QDs with high crystallinity. The results showed that the d-spacing of both 2D perovskite QDs with *n* = 1 was estimated to be 0.27 nm, which matches to the (0100) phase. The d-spacing of 2D Br series and I series perovskite QDs with *n* = 2 was calculated to be ~ 0.29 nm and ∼0.69 nm, which is related to the (200) and (111) plane of 2D perovskite QDs, respectively.

**Fig. 4:**
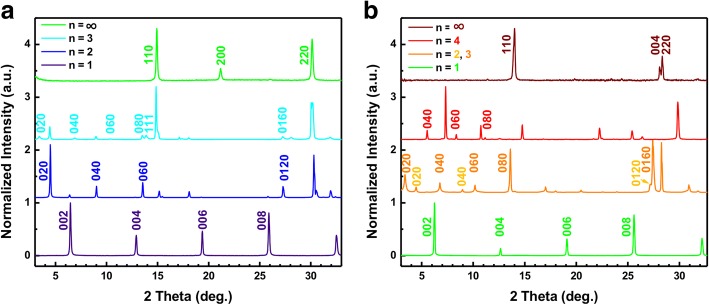
XRD spectra of 2D RP perovskite QDs with **a** Br series and **b** I series

To investigate the layered crystalline structures of these 2D Br series and I series perovskite QDs, XRD patterns were performed as shown in Fig. 4. The results indicate that additional low angle peaks found for each 2D perovskite QDs are attributed to the incremental expansion of unit cell of perovskite with increases of the thickness of the 2D perovskite layers in the crystal structure. All the 2D perovskite QDs with *n* ≥ 2 compositions show diffraction peaks at 15.1° and 14.1° for Br series and I series, respectively, which are the same as the (100) diffraction patterns of the 3D perovskite materials [33, 34]. The peak in the two series is more broadened with increases of *n* value, indicating that the grain size of 2D perovskite QDs become smaller than that of 3D MAPbBr_3_ [35]. Also, the angle of (100) phase in Br series is lower than that of I series, which can be attributed to the smaller ionic radius of the Br series compared with I—that the lattice parameter. Besides, a series of Bragg reflections peaks at lower angles (2*θ* < 14.1°) are observed for 2D I series perovskites QDs (Fig. 4b). This indicates that the large BA group is incorporated into the perovskite crystal structure, resulting in enlarging the size of unit cells in comparison with 3D perovskite [36, 37]. We also found a series of reflections peak appeared at low angles (2*θ* < 14°) for these 2D RP perovskite QDs. In Br series of 2D RP perovskite QDs, the diffraction peaks (2*θ* < 14°) are attributed to the *n* = 1, *n* = 2, and *n* = 3 phases, but no diffraction patterns from *n* ≥ 4 phases are observed, which is similar to 3D perovskite NCs. For I series, there are *n* = 1, *n* = 2, *n* = 3, and *n* = 4 phases found in the diffraction peaks. In both series, only 2D RP perovskite QDs with *n* = 1 value has a single phase that exists. For other *n* value compositions, there are usually two phases presented in the synthesized sample. All phases for different *n* values have been pointed out in both XRD spectra. According to the Scherrer equation, the estimated diameter of the QDs is similar to the obtained result from TEM images.

**Fig. 5:**
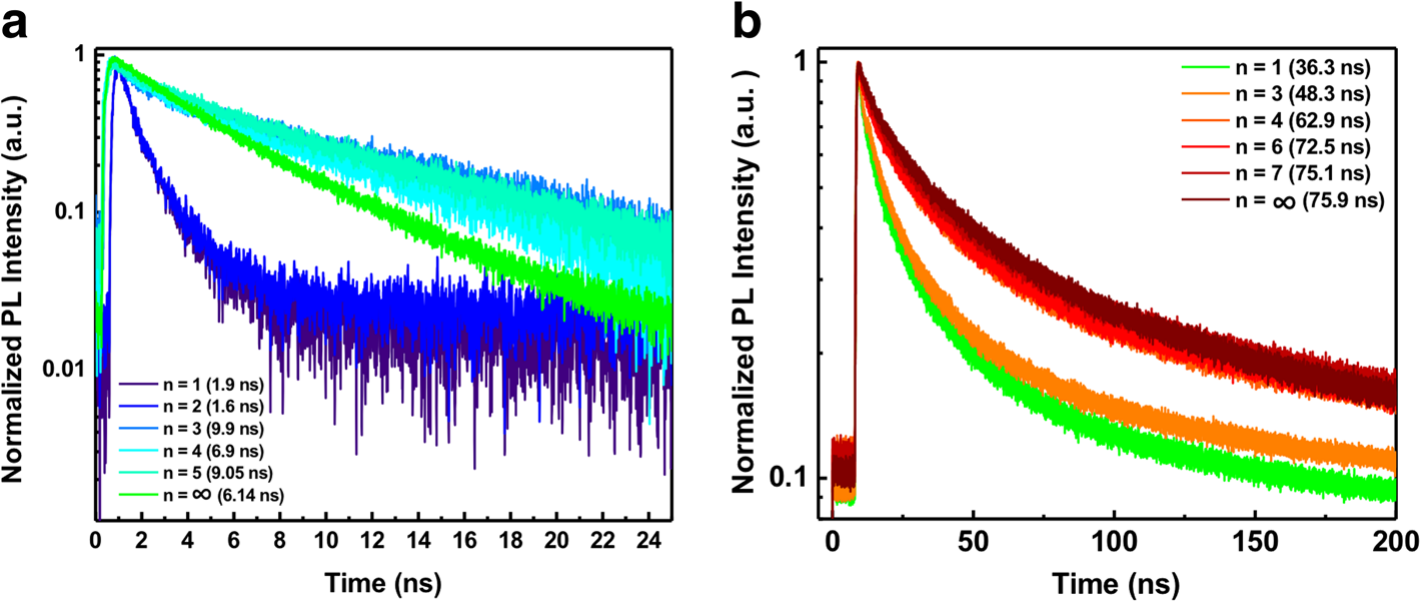
Time-resolved PL decay of 2D RP perovskite QDs with **a** Br series and **b** I series by using pulse laser with a wavelength of 375 nm and 466 nm, respectively

To investigate the photophysical property of these 2D Br series and I series perovskite QDs, time-resolved PL spectroscopy (TRPL) was performed as shown in Fig. 5. The results show non-exponential decay traces with average lifetimes of *τ* = 1 ~ 9 ns and 48 ~ 75 ns for Br series and I series, respectively. It is found that I series QDs with red emission show higher PL decay time than Br series QDs due to the smaller band gap of I series. Moreover, our 2D I series QDs demonstrate a relatively longer lifetime in comparison with exfoliated (BA)_2_(MA)_*n* − 1_Pb_*n*_I_3*n* + 1_ crystal (*τ* < 10 ns) reported in the literature [11, 38]. Overall results indicate that 2D perovskite QDs exhibit a fewer trap state resulting in less nonradiative decay mechanisms such as electron–phonon coupling and long PL lifetime.

## Conclusions

A facile synthesis method of highly luminescent 2D RP perovskite QDs with Br series and I series has been illustrated. The band gap of the QDs for Br series and I series can be adjusted by the ratio of MA and halide, expressing tunable emission light across the visible region 410 to 523 nm and 527 to 761 nm, respectively. A remarkably high quantum yield of up to 48.6% was obtained. Also, we found that 2D perovskite QDs exhibited more optical stability in comparison with 3D perovskite QDs, which resulted in less nonradiative decay from electron–phonon coupling. It is believed that the brightly luminescent 2D perovskite QDs will be the trigger for developing much more stable solution-processed perovskite materials in optoelectronic applications.

## Methods

### Chemicals used

Lead (II) bromide (98 + %, Acros), lead (II) iodide (99%, Acros), methylamine solution (33 wt% in absolute ethanol, Acros), n-butylamine (99.5%, Acros), hydrobromic acid (48%, Fisher), hydroiodic acid (57 wt% aqueous, Acros), octylamine (99 + %, Acros), oleic acid (SLR grade, Alfa Aesar), *N*,*N*-dimethylformamide (99.8%, Macron), and toluene (HPLC quality, Acros). All reagents and solvents were used as received without further purification.

### Synthesis of alkylammonium halide

Butylammonium bromide (BABr), methylammonium bromide (MABr), butylammonium iodide (BAI), and methylammonium iodide (MAI) were prepared by addition of HBr (48%) or HI (57%) to butylamine (99.5%) or to a solution of methylamine (33 wt%) in absolute ethanol, respectively. The molar ratio of the acid and the amine was 1.1:1.0. The resulting mixture was stirred for 2 h and maintained at 0 °C using an ice water bath. Then, the solvent was removed by a rotary evaporator. The precipitate was washed with diethyl ether by stirring the solution for 30 min several times. After filtration, the white solid was dried at 60 °C in a vacuum oven. After drying overnight, the alkylammonium halide crystals were all sealed under argon and transferred into a glove box for further use.

### 2D-layered nanocrystals (NCs) synthesis

All syntheses were carried out at room temperature under ambient conditions. For different layered 2D NCs, BAX, MAX, and PbX_2_ (X = Br or I) were mixed in different molar ratios (2: *n* − 1: 3*n* + 1, *n* = 1, 2, 3, …, ∞) and were dissolved in DMF forming 0.04 mM PbX_2_ solution. 0.5 mL OA and 0.05 mL octylamine were added into 5 mL of the solution. Next, 100 μL of this mixture was injected into 10 ml toluene under vigorous stirring to form 2D NCs. Detailed synthesis compositions were presented in Fig. 1b.

### Characterizations

The morphology and structure of 2D perovskites QDs were revealed by transmission electron microscope (TEM) and high-resolution TEM, respectively. TEM images were conducted in a 200 kV transmission electron microscope (JEOL, 2100F) and 120 kV transmission electron microscope (HITACHI, HT7700). The crystal structures and qualities of 2D perovskites QDs were determined from the XRD *θ*–2*θ* scan data by using powder X-ray diffractometer (Rigaku Miniflex 600). Photoluminescence spectra were obtained from fluorescence spectrophotometer (HITACHI F-4500). The PLQY of 2D RP perovskite (BA)_2_(MA)_*n* − 1_Pb_*n*_X_3*n* + 1_ QDs were measured in the toluene, using C-102 and DCJTB compounds as the standards. The QY of C-102 and DCJTB are 0.76 and 0.78, respectively [39, 40]. Time-resolved photoluminescence (TRPL) spectroscopy was acquired using a time-correlated single photon counting (TCSPC) spectrometer set-up (FluoTime 300, PicoQuant GmbH). Samples were photo-excited using a 375 nm and 466 nm laser head (LDH-P-C-470, PicoQuant GmbH) with a pulse duration of 70 ps, fluence of 90 μW and a repetition rate of 4 MHz.

## Additional file

Additional file 1: Additional TEM images and absorption spectra. (DOCX 2168 kb)

## Abbreviations

(BA)_2_(MA)_*n* − 1_Pb_*n*_Br_3*n* + 1_: Br series
(BA)_2_(MA)_*n* − 1_Pb_*n*_I_3*n* + 1_: I series
2D: Two-dimensional
3D: Three-dimensional
BA: 1-Butylammonium
BAX: Butylammonium halogen
CB: Chlorobenzene
DMF: Dimethylformamide
FWHM: Full width at half maximum
HRTEM: High-resolution TEM
MA: Methylammonium
MAX: Methylammonium halogen
OA: Oleic acid
OLA: Octylamine
PL: Photoluminescence
PLQYs: Photoluminescence quantum yields
QDs: Quantum dots
RP: Ruddlesden–Popper
TEM: Transmission electron microscopy
TRPL: Time-resolved PL spectroscopy
XRD: X-ray diffraction
