# Supplementary material for: Facile synthesis of two-dimensional Ruddlesden–Popper perovskite quantum dots with fine-tunable optical properties
Source: Nanoscale Res Lett. 2018 Aug 22;13:247. doi: 10.1186/s11671-018-2664-5 (PMC6104471; doi:10.1186/s11671-018-2664-5)
Supplement: Supplementary file 1 — Additional TEM images and absorption spectra. (DOCX 2168 kb) [file 11671_2018_2664_MOESM1_ESM.docx]

Supporting Information

**Facile Synthesis of Two-dimensional Ruddlesden Popper Perovskite Quantum Dots with Fine Tunable Optical Properties**

Yi-Hsuan Chang, ^1#^ Jou-Chun Lin,^1,2#^ Yi-Chia Chen,^1^ Tsung-Rong Kuo,^2^* Di-Yan Wang^1^*


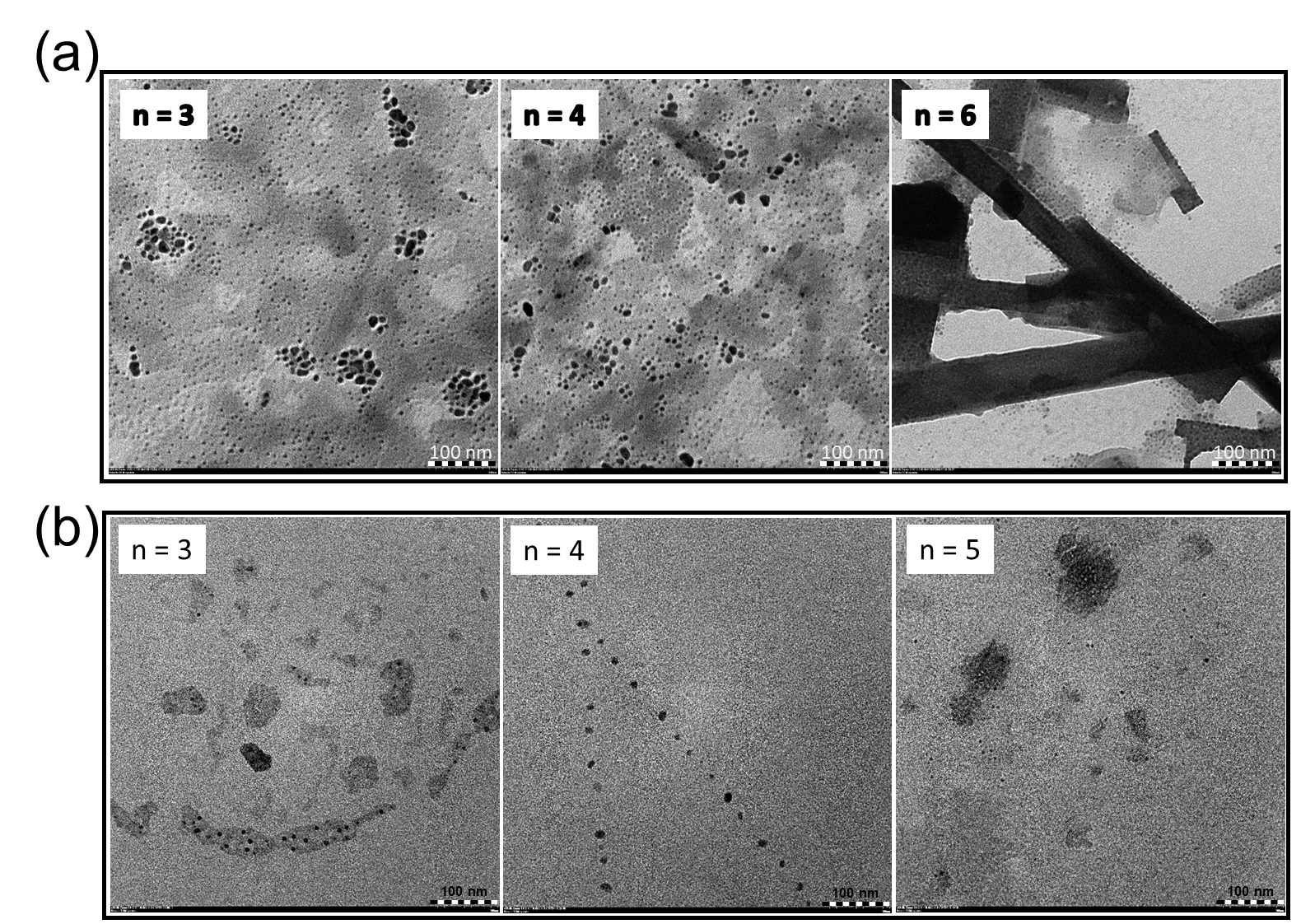


**Figure S1.** (a) TEM images of 2D Br-series perovskite QDs with n= 3,4 and 6. (b) TEM images of 2D I-series perovskite QDs with n= 3, 4 and 5.


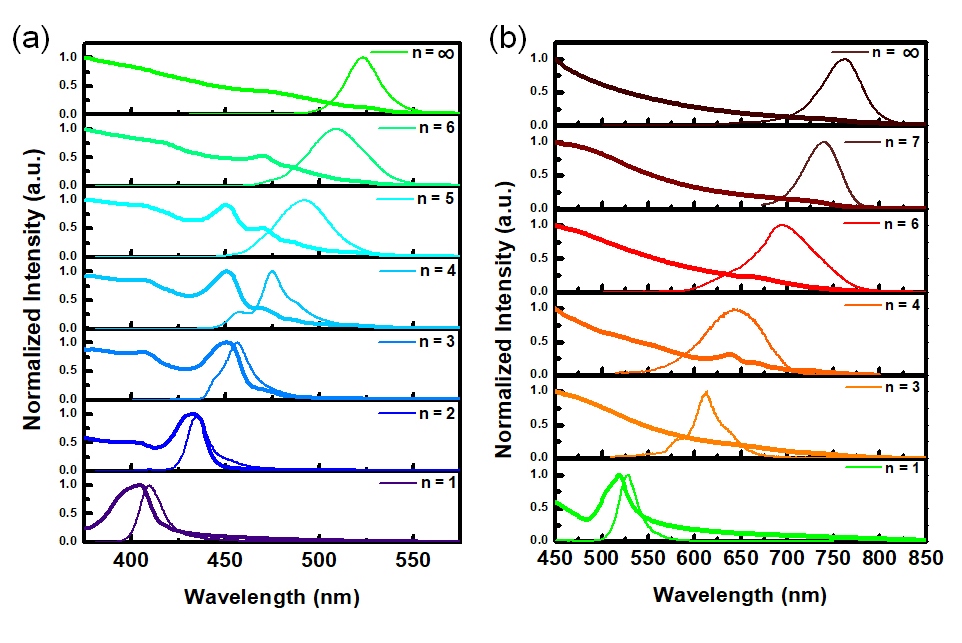
**Figure S2.** The UV-Vis absorption spectra and related PL spectra of 2D RP perovskite QDs with (a) Br-series and (b) I-series with varied n values.
